# Mice Abundant in Muricholic Bile Acids Show Resistance to Dietary Induced Steatosis, Weight Gain, and to Impaired Glucose Metabolism

**DOI:** 10.1371/journal.pone.0147772

**Published:** 2016-01-29

**Authors:** Ylva Bonde, Gösta Eggertsen, Mats Rudling

**Affiliations:** 1 Department of Medicine, Karolinska Institute, Karolinska University Hospital Huddinge, Stockholm, Sweden; 2 Department of Biosciences and Nutrition, Karolinska Institute, Karolinska University Hospital Huddinge, Stockholm, Sweden; 3 Department of Laboratory Medicine, Karolinska Institute, Karolinska University Hospital Huddinge, Stockholm, Sweden; East Tennessee State University, UNITED STATES

## Abstract

High endogenous production of, or treatment with muricholic bile acids, strongly reduces the absorption of cholesterol. Mice abundant in muricholic bile acids may therefore display an increased resistance against dietary induced weight gain, steatosis, and glucose intolerance due to an anticipated general reduction in lipid absorption. To test this hypothesis, mice deficient in steroid 12-alpha hydroxylase (*Cyp8b1*^*-/-*^) and therefore abundant in muricholic acids were monitored for 11 weeks while fed a high fat diet. Food intake and body and liver weights were determined, and lipids in liver, serum and feces were measured. Further, responses during oral glucose and intraperitoneal insulin tolerance tests were evaluated.

On the high fat diet, *Cyp8b1*^*-/-*^ mice displayed less weight gain compared to wildtype littermates (*Cyp8b1*^*+/+*^). In addition, liver enlargement with steatosis and increases in serum LDL-cholesterol were strongly attenuated in *Cyp8b1*^*-/-*^ mice on high fat diet. Fecal excretion of cholesterol was increased and there was a strong trend for doubled fecal excretion of free fatty acids, while excretion of triglycerides was unaltered, indicating dampened lipid absorption. On high fat diet, *Cyp8b1*^*-/-*^ mice also presented lower serum glucose levels in response to oral glucose gavage or to intraperitoneal insulin injection compared to *Cyp8b1*^*+/+*^.

In conclusion, following exposure to a high fat diet, *Cyp8b1*^*-/-*^ mice are more resistant against weight gain, steatosis, and to glucose intolerance than *Cyp8b1*^*+/+*^ mice. Reduced lipid absorption may in part explain these findings. Overall, the results suggest that muricholic bile acids may be beneficial against the metabolic syndrome.

## Introduction

Bile acids (BAs) are crucial for an efficient intestinal absorption of lipids and lipophilic compounds. BAs also have metabolic effects on triglyceride (TG) and glucose metabolism, elicited by interaction with the farnesoid X receptor (FXR) and the G protein-coupled BA receptor 1, (Gpbar1 or TGR-5) [1]. There are differences in BA metabolism between mice and humans. One such important difference is that mice are capable of synthesizing muricholic BAs (MCAs), a group of hydrophilic BAs that are hydroxylated at the 6-position. The synthetic pathways and the physiological functions of the MCAs are unclear. Feeding mice α-, β- or ω-MCAs strongly reduces the absorption of cholesterol from about 35% down to 12–18%, while feeding cholic acid increases the absorption level up to >60% [2] and concomitantly reduces MCAs in the liver by 80–90% [3, 4]. Mice deficient in steroid 12-alpha hydroxylase (*Cyp8b1*^*-/-*^) cannot synthetize cholic acid; therefore more chenodeoxycholic acid is made which is a precursor of MCAs [5], leading to an accumulation of MCAs in these mice [6]. The *Cyp8b1*^*-/-*^ mice are further characterized by a reduced absorption of cholesterol (23%) as compared to *Cyp8b1*^*+/+*^ (54%) [7]. The *Cyp8b1*^*-/-*^ phenotype also includes an increased BA synthesis, an increased intestinal expression of apical sodium-dependent BA transporter, and an enlarged BA pool [3]. This phenotype is shared with several other mouse models such as germ-free, fibroblast growth factor receptor 4-deficient, and antibiotic-treated mice [3, 8]. Interestingly, these three mouse models are all resistant to dietary induced glucose intolerance [9–12]. We hypothesized that the presence of high levels of MCAs would be important for this resistance and that, accordingly, *Cyp8b1*^*-/-*^ mice should be resistant to glucose intolerance as well. This would be due to an anticipated MCA-induced general reduction in fat absorption, in line with previous reports on cholesterol and fat absorption in these mice of about 20–40% [6, 7]. To investigate this, *Cyp8b1*^*-/-*^ and *Cyp8b1*^*+/+*^ mice of both genders were challenged with a high fat diet (HFD) or chow for 11 weeks. The results show that *Cyp8b1*^*-/-*^ mice are indeed more resistant against HFD-induced impaired glucose metabolism. *Cyp8b1*^*-/-*^ mice also display strong resistance against weight gain, hepatomegaly, steatosis, and hypercholesterolemia. The results also indicate that a reduced intestinal absorption of lipids may be involved in the mechanisms behind these effects. Overall, these data indicate that MCAs may improve glucose intolerance, steatosis, and overweight.

## Materials and Methods

### Animals

In this study, 10–11 months old *Cyp8b1*^*-/-*^ mice and their *Cyp8b1*^*+/+*^ littermates, inbred as described [6], were used. Altogether, 73 mice were divided into the following groups; *Cyp8b1*^*+/+*^ mice fed regular mouse chow (wt chow; 8 females and 10 males), *Cyp8b1*^*-/-*^ mice fed regular mouse chow (ko chow; 10 females and 9 males), *Cyp8b1*^*+/+*^ mice fed a HFD (wt HFD; 8 females and 10 males), and *Cyp8b1*^*-/-*^ mice fed HFD (ko HFD; 9 females and 9 males). The HFD was obtained from Research Diets Inc. (D12492, New Brunswick, NJ) and contained 60 kcal% fat. The regular mouse chow (RM3 (P), Special Diet Services, Scanbur, Stockholm, Sweden) contained 11 kcal% fat. Mice were kept in a temperature-controlled, pathogen-free environment with a 12 hour light to dark cycle with food and water *ad libitum*. Body weight (individual measurements) and food intake (groupwise) were monitored weekly.

### Animal procedures

After 9 weeks, an oral glucose tolerance test (OGTT) was performed on 4h-fasted mice by giving an oral gavage of glucose (1 g glucose/kg BW, G8769, Sigma-Aldrich, St. Louis, MO). After 10 weeks, an intraperitoneal insulin tolerance test (IITT) was performed on 4h-fasted mice by administering intraperitoneal injections of insulin (0.75 U insulin/kg BW, Actrapid Penfill, Novo Nordisk, Bagsvaerd, Denmark). Blood glucose was assayed in blood samples taken from the tip of the tail before glucose or insulin were administered (at 0 min), and again at 15, 30, 60, and 130 minutes after administration and analyzed using an ACCU-CHEK Aviva and test strips (Roche, Mannheim, Germany). After 11 weeks of treatment, blood was collected from 4h-fasted mice by cardiac puncture during anesthesia with Isoflurane, after which the anesthetized mice were sacrificed by cervical dislocation. Collected organs were weighted and flash-frozen in liquid nitrogen. The Stockholm South Ethical Committee approved this research (Approval number S96-11).

### Hepatic total cholesterol

Hepatic total cholesterol levels were assayed in 20% homogenates prepared from 0.3 g of liver for each individual. 1 mL of chloroform:methanol 2:1 and 0.1 mL of 0.9% sodium chloride were added to 20 μL of homogenate in duplicates that were vortexed and left at room temperature to separate for 1 hour. The lower phase was collected and placed in 60°C under a stream of liquid nitrogen to evaporate prior to being re-dissolved in 1 mL of chloroform:methanol 2:1 of which 0.5 mL was collected, evaporated, and 1 mL of 0.5 M potassium hydroxide in ethanol added before samples were placed in 70°C for 90 minutes to re-dissolve. Then, 1 mL of water and 5 mL of hexane were added and samples shaken vigorously prior to being left at room temperature to separate for 1 hour. The upper phase was collected and placed in 60°C under a stream of liquid nitrogen to evaporate prior to being silylated with pyridine/hexametyldisilazane/chlorotrimetylsilane (3:2:1, v/v/v), evaporated, and re-dissolved in 0.1 mL of hexane. Samples were analyzed using gas chromatography-mass spectrometry using ^2^H_7_-cholesterol as an internal standard. Total cholesterol content in the liver was calculated for each individual by multiplying the cholesterol concentration by the liver mass.

### Hepatic total TG

Hepatic total TG levels were assayed in homogenates of 0.2 g liver in 4 mL of chloroform:methanol 2:1 for each individual. Homogenates were centrifuged at 3500 rpm for 10 min at room temperature. 2 mL of the supernatant was collected and 0.8 mL of 0.9% sodium chloride was added before samples were centrifuged at 3500 rpm for 10 min at room temperature. 0.6 mL of the lower phase was collected in duplicates and evaporated prior to addition of 0.6 mL methanol with 0.05% Tween 80 and vortexed. Samples were sonicated, placed in 50°C, and vortexed until dissolved. A Tecan Infinite® M200 reader (Männedorf, Switzerland) and reagents from Roche Diagnostics GmbH (Mannheim, Germany) were used to analyze samples according to the manufacturer´s instructions. Total TG content in the liver was calculated for each individual by multiplying the cholesterol concentration by the liver mass.

### Lipoprotein cholesterol

Lipoprotein cholesterol within VLDL, LDL, and HDL lipoprotein fractions were measured by fast performance liquid chromatography [13] using reagents from Roche Diagnostics GmbH.

### Serum campesterol

Serum campesterol levels were determined in duplicate samples using gas chromatography-mass spectrometry as described [14] and normalized for serum cholesterol levels. This plant sterol serves as an indirect marker of cholesterol absorption [15].

### Fecal free fatty acids, cholesterol and triglycerides

Fecal free fatty acids (FFAs), cholesterol and triglycerides (TGs) were measured in 24h-feces collections from each of the 12 cages of HFD-fed mice (6 cages/group). Collections were dried in an incubator until being weight stable. Each fecal collection was then grinded to a homogenous powder of which duplicates of 0.1 g (FFAs) or 0.5 g (cholesterol and TGs) were subjected to lipid extraction with Folch. For analysis of FFAs, samples were centrifuged at 3000 rpm for 10 min at room temperature. The chloroform phase was collected and 0.2 mL was allowed to dry prior to lipids being re-dissolved in 400 μL PBS containing 0.05% Triton X-100. Samples were then analyzed with reagents from Wako Chemicals Gmbh (NEFA-HR(2), Neuss, Germany) according to the manufacturers’ instructions using a Tecan Infinite® M200 reader. For analysis of cholesterol and TGs, samples were centrifuged at 3000 rpm for 10 min at room temperature and the chloroform phase was collected and dried prior to being re-dissolved in 600 μL methanol containing 0.05% Tween 80. 1 mL of reagent (Triglycerides GPO-PAP, or Cholesterol CHOD-PAP, respectively, Cobas, Roche Diagnostics GmbH, Mannheim, Germany) was added to 60 μL of sample and incubated in water bath (+37°C) for 15 minutes prior to analyzed using a Tecan Infinite® M200 reader.

### Total glucagon-like peptide-1 (GLP-1)

Total glucagon-like peptide-1 (GLP-1) levels were assayed in duplicate serum aliquots by an electrochemiluminescence immunoassay according to the manufacturers’ guidelines (K150JVC-2, Meso Scale Discovery (MSD), Gaithersburg, MD) using a SECTOR Imager 6000 instrument (MSD).

### Statistical analyses

Data show mean ± standard error of mean (SEM). Significances of differences between groups were tested by 1-way ANOVA followed by post-hoc comparisons according to Tukey’s test, using GraphPad Prism software (GraphPad Software Inc. San Diego, CA). Significances of differences between groups regarding fecal lipid excretion were tested by t-test.

## Results

### *Cyp8b1*^*-/-*^ mice on HFD display an enhanced protection against glucose intolerance

**Fig 1:**
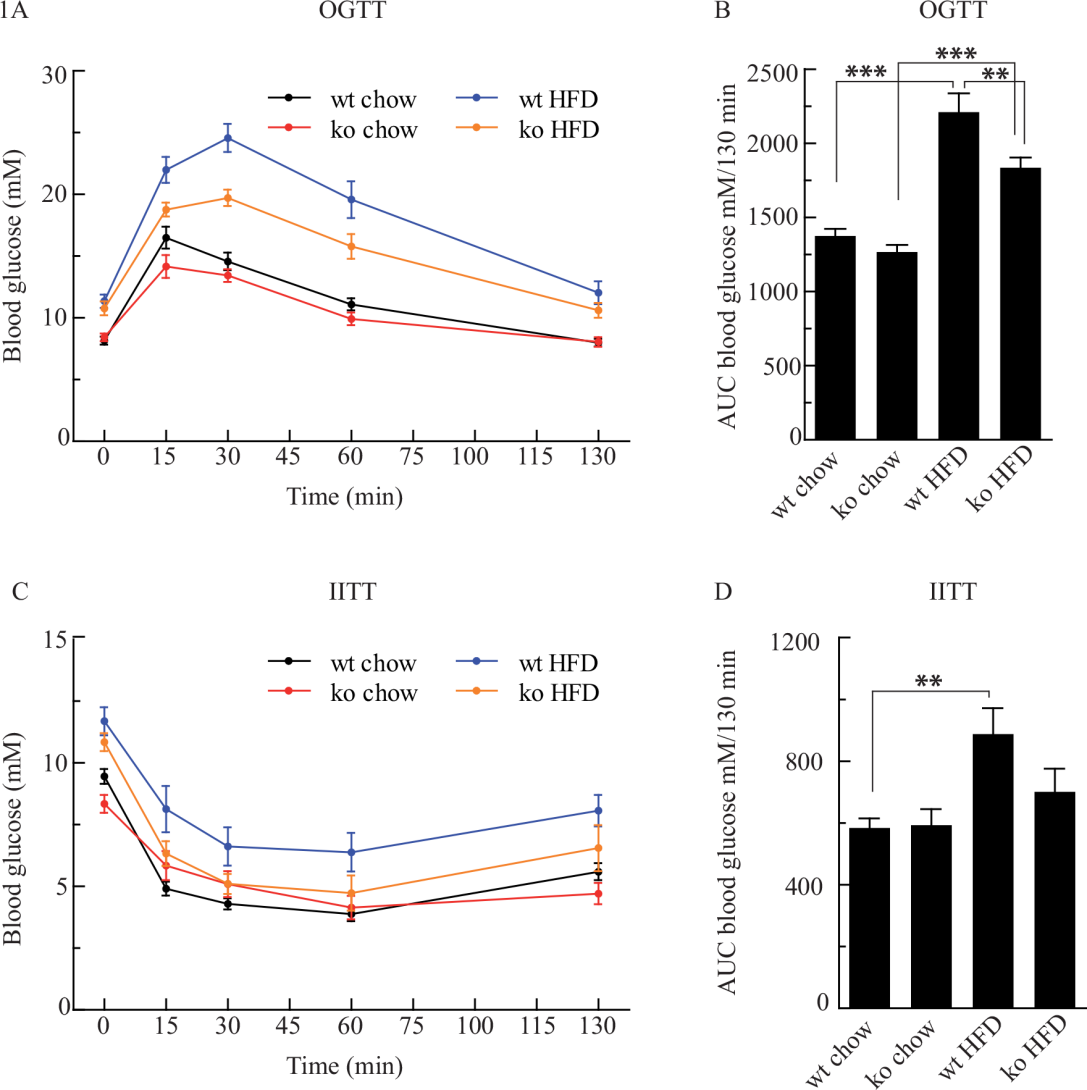
Cyp8b1^-/-^ (ko) mice show improved glucose tolerance and response to insulin on a high fat diet (HFD) as compared to Cyp8b1^+/+^ (wt) mice. Blood glucose levels in response to oral glucose tolerance test (OGTT) **(A)** and area under the curves (AUCs) **(B)**. Blood glucose levels in response to intraperitoneal insulin tolerance test (IITT) **(C),** and as AUCs **(D)**. Data is shown as mean ±SEM, *n* = 16 for all groups. *** = p<0.001 and ** = p<0.01.

After 9 weeks of feeding with chow or with HFD an OGTT (Fig 1A) showed that *Cyp8b1*^*+/+*^ and *Cyp8b1*^*-/-*^ mice fed chow are equally tolerant to glucose, presented as area under the curve (AUC) in Fig 1B, although there was a trend for lower glucose levels in *Cyp8b1*^*-/-*^ mice. In both *Cyp8b1*^*+/+*^ and *Cyp8b1*^*-/-*^ mice fed HFD, glucose tolerance was impaired as compared to their respective chow-fed controls. However, this impairment was significantly less severe in *Cyp8b1*^*-/-*^ mice as compared to *Cyp8b1*^*+/+*^ mice. An IITT performed after 10 weeks of treatment (Fig 1C) showed that chow-fed *Cyp8b1*^*+/+*^ and *Cyp8b1*^*-/-*^ mice respond equally well to insulin, presented as AUC in Fig 1D. *Cyp8b1*^*+/+*^ fed HFD displayed an impaired response to insulin as compared to its chow-fed control, while the response to insulin in *Cyp8b1*^*-/-*^ mice fed HFD did not differ from that of its chow-fed control.

### *Cyp8b1*^*-/-*^ mice fed HFD are protected against hepatomegaly and steatosis and display improved lipoprotein profiles

**Fig 2:**
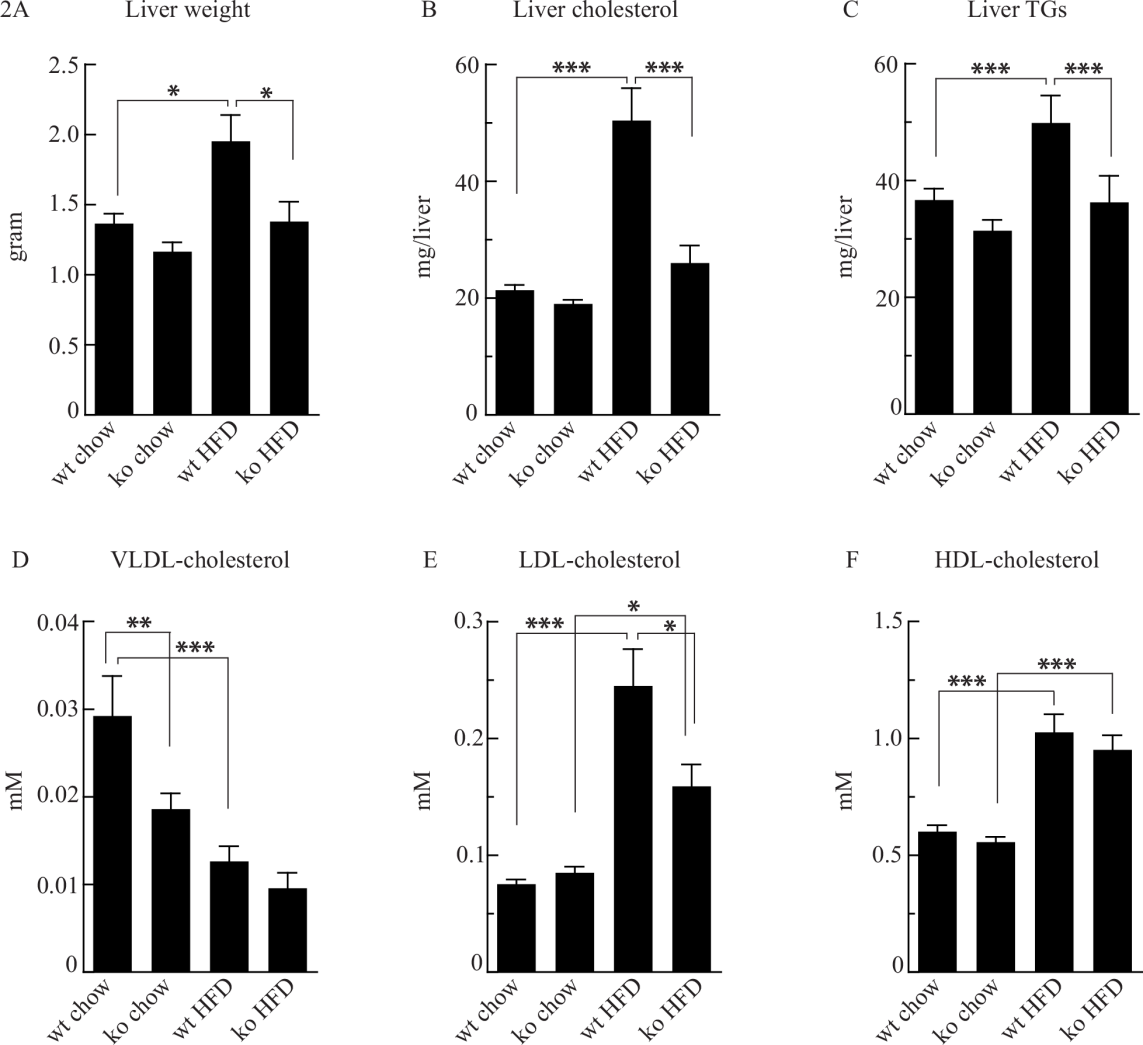
Cyp8b1^-/-^ (ko) mice are protected from liver hypertrophy and fatty liver when exposed to high fat diet (HFD) and display improved lipoprotein profiles as compared to Cyp8b1^+/+^ (wt) mice. Liver weight **(A)**, liver cholesterol **(B)** and liver triglycerides (TGs) **(C)**. Serum cholesterol in VLDL **(C)**, LDL **(D)**, and HDL **(E)** lipoprotein fractions. Data is shown as mean ±SEM, *n* = (18–19) for all groups. *** = p<0.001, ** = p<0.01, and * = p<0.05.

There was no difference in liver weight between *Cyp8b1*^*+/+*^ and *Cyp8b1*^*-/-*^ mice fed chow (Fig 2A). In *Cyp8b1*^*+/+*^ mice fed HFD, liver weight increased 43% while liver weight in *Cyp8b1*^*-/-*^ mice fed HFD was unaltered. Comparison of liver weight between *Cyp8b1*^*+/+*^ and *Cyp8b1*^*-/-*^ mice fed HFD revealed that livers from the latter were 29% lighter.

The contents of cholesterol and TGs harbored in the liver were similar in *Cyp8b1*^*+/+*^ and *Cyp8b1*^*-/-*^ mice fed chow (Fig 2B and 2C). In *Cyp8b1*^*+/+*^ mice fed HFD, cholesterol and TG contents were increased 43% and 36% respectively while unaltered in *Cyp8b1*^*-/-*^ mice fed HFD as compared to their respective chow-fed controls. In fact, *Cyp8b1*^*-/-*^ mice on HFD had 25% lower cholesterol and 27% lower TG content than *Cyp8b1*^*+/+*^ mice on HFD. Serum LDL- and HDL-cholesterol levels did not differ between chow-fed *Cyp8b1*^*+/+*^ and *Cyp8b1*^*-/-*^ mice, while basal VLDL-cholesterol was 36% lower in *Cyp8b1*^*-/-*^ mice (Fig 2D–2F). In *Cyp8b1*^*+/+*^ mice on HFD, LDL- and HDL-cholesterol were increased 225% and 71% respectively, as compared to its chow-fed control. Also in *Cyp8b1*^*-/-*^ mice on HFD, LDL- and HDL-cholesterol were increased 87% and 71%, respectively, as compared to its chow-fed control. However, LDL-cholesterol in *Cyp8b1*^*-/-*^ mice on HFD was 35% lower than in the *Cyp8b1*^*+/+*^ mice on HFD, while HDL-cholesterol levels were similar.

### Body weight gain and food efficiency are reduced in *Cyp8b1*^*-/-*^ mice on HFD

**Fig 3:**
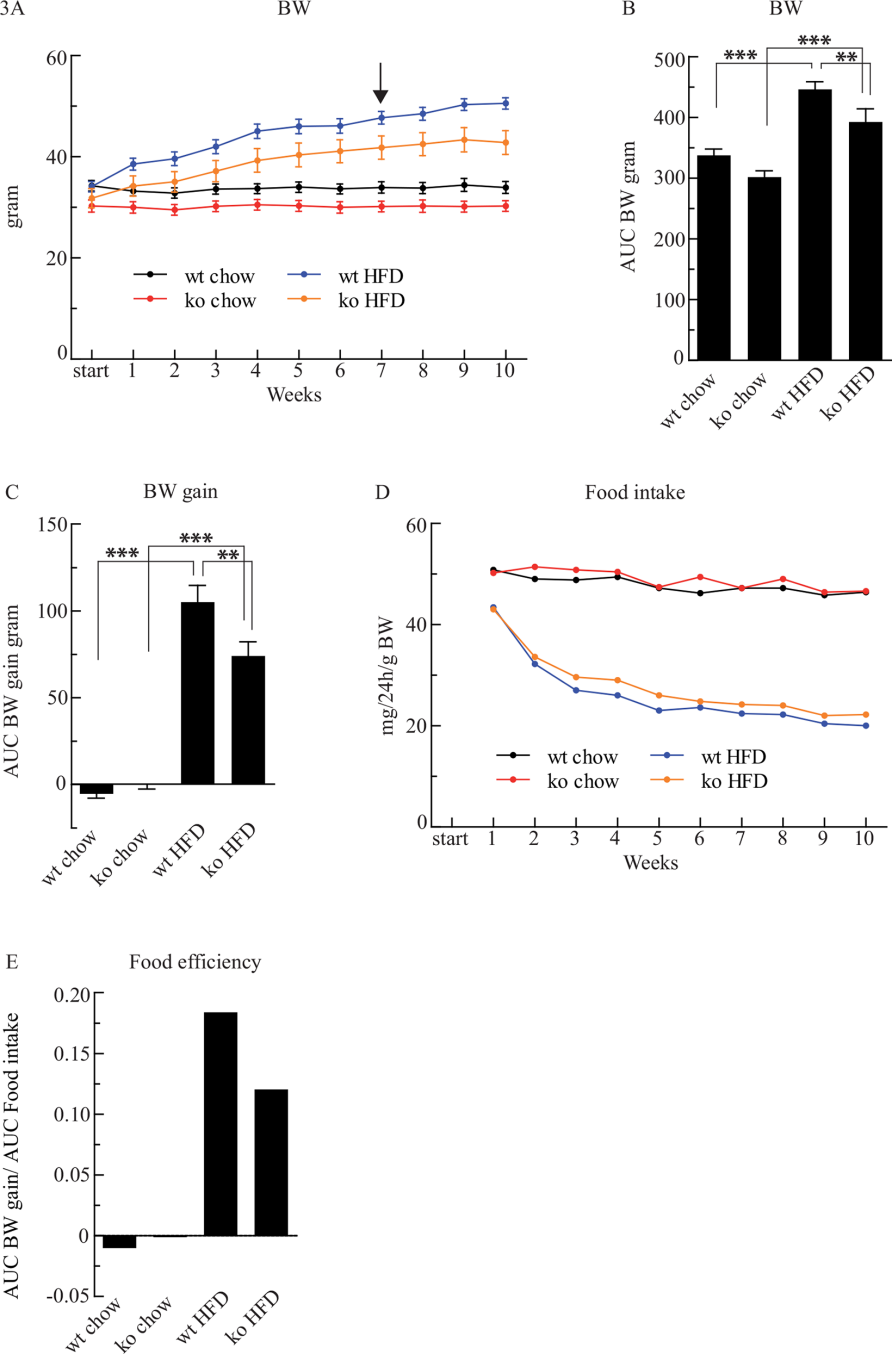
Cyp8b1^-/-^ (ko) mice show improved resistance to body weight (BW) gain following exposure to a high fat diet (HFD) and display lowered food efficiency index than Cyp8b1^+/+^ (wt) mice. Body weight **(A)** and area under the curve (AUC) **(B)**. The arrow denotes the time point at which a significant difference in body weight between *Cyp8b1*^*-/-*^ and *Cyp8b1*^*+/+*^ mice on HFD first occurred, and this difference persisted throughout the experiment. Body weight gain shown as AUC **(C)**, food intake **(D)** and food efficiency **(E)**. Where indicated, data is shown as mean ±SEM, *n* = (17–19) for all groups. *** = p<0.001, ** = p<0.01, and * = p<0.05.

Body weight did not differ between *Cyp8b1*^*+/+*^ and *Cyp8b1*^*-/-*^ mice on chow throughout the experiment (Fig 3A and 3B) although *Cyp8b1*^*-/-*^ showed a strong trend to be tended to be lighter. Significantly increased body weights were found in both *Cyp8b1*^*+/+*^ and *Cyp8b1*^*-/-*^ mice fed HFD but body weight gain was 30% lower in *Cyp8b1*^*-/-*^ mice than in *Cyp8b1*^*+/+*^ mice, as shown in Fig 3C. Food intake was similar in chow-fed *Cyp8b1*^*+/+*^ and *Cyp8b1*^*-/-*^ mice, as well as in both *Cyp8b1*^*+/+*^ and *Cyp8b1*^*-/-*^ mice on HFD (Fig 3D). The HFD significantly reduced the amount of food consumed, but calculations of total caloric intake showed that the caloric intake was similar to that of the chow-fed controls. The mean AUC of body weight gain divided by the mean AUC of food intake (referred to as food efficiency, Fig 3E) indicated that *Cyp8b1*^*-/-*^ mice on HFD were less efficient in utilizing the food consumed to gain body weight than were the *Cyp8b1*^*+/+*^ mice on HFD.

### Fecal excretion of FFAs, cholesterol, and TGs, and serum GLP-1 levels in *Cyp8b1*^*-/-*^ mice

**Fig 4:**
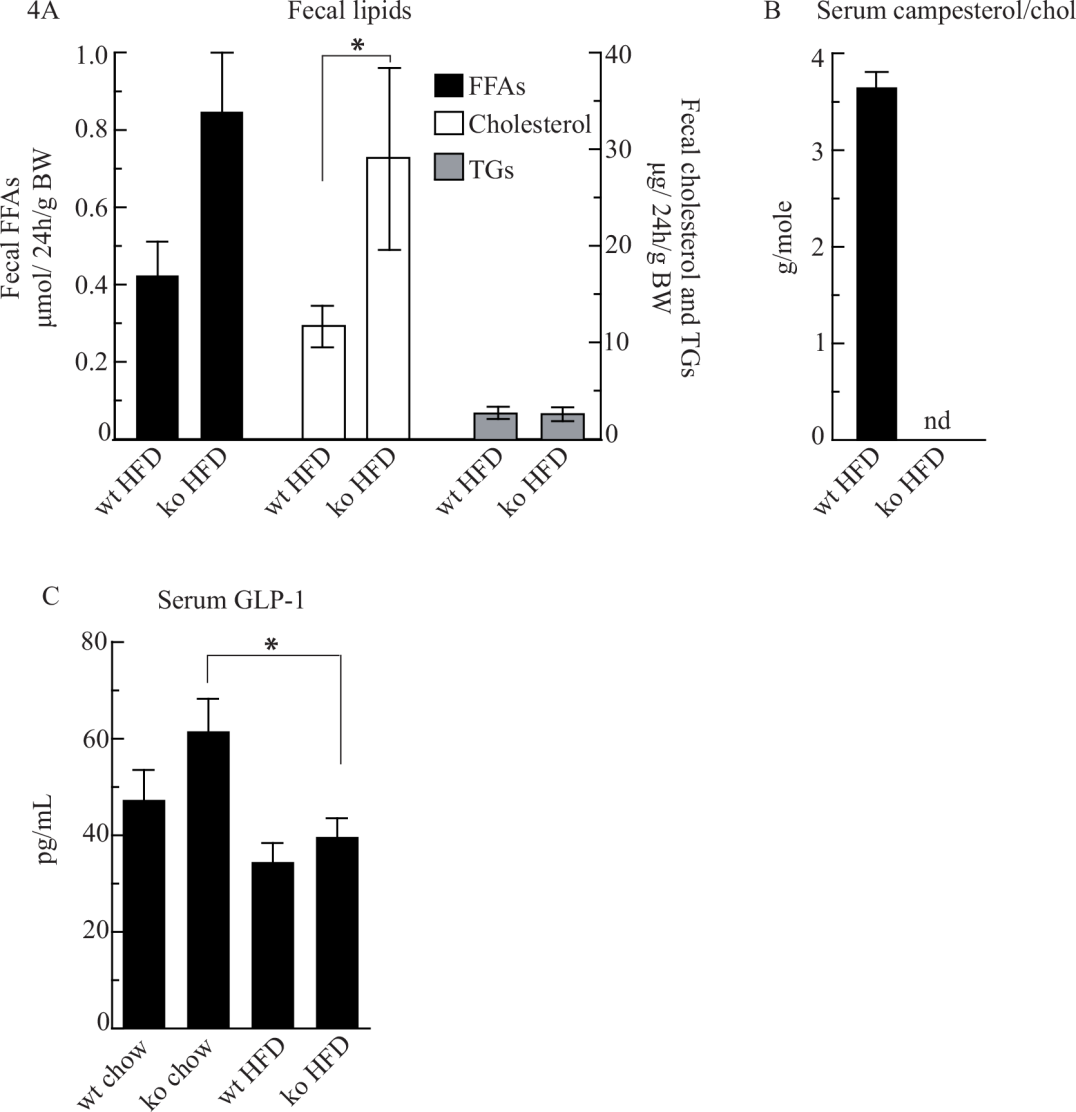
Cyp8b1^-/-^ (ko) mice have increased fecal excretion of cholesterol and free fatty acids (FFAs) when fed a high fat diet (HFD) as compared to Cyp8b1^+/+^ (wt) mice. Fecal excretion of FFAs, cholesterol, and triglycerides **(A)**, serum campesterol levels, a marker of cholesterol absorption **(B)**, and serum glucagon-like peptide-1 (GLP-1) levels **(C)**. Where indicated, data is shown as mean ±SEM, *n* = (17–19) for all groups. *** = p<0.001, ** = p<0.01, and * = p<0.05.

Excretion of fecal cholesterol in mice on HFD was more than doubled in *Cyp8b1*^*-/-*^ mice compared to that in *Cyp8b1*^*+/+*^ mice (Fig 4A). In line with this, serum levels of campesterol, commonly used to evaluate intestinal cholesterol absorption, were strongly reduced in *Cyp8b1*^*-/-*^ mice on HFD, with levels below the detection limit (Fig 4B). Also fecal excretion of FFAs was doubled in *Cyp8b1*^*-/-*^ mice on HFD although did not reach statistical significance. Fecal excretion of TGs was unaltered in *Cyp8b1*^*-/-*^ mice on HFD as compared to *Cyp8b1*^*+/+*^ mice on HFD.

Finally, assay of serum levels of GLP-1 revealed a trend for increased serum GLP-1 levels in chow-fed *Cyp8b1*^*-/-*^ mice (Fig 4C). On the HFD, GLP-1 levels were reduced by 37% in the *Cyp8b1*^*-/-*^ mice and there was a strong trend for reduced GLP-1 levels also in *Cyp8b1*^*+/+*^ mice.

## Discussion

Several mouse models that are resistant to dietary induced glucose intolerance [9–12] share phenotypic traits with *Cyp8b1*^*-/-*^ mice e.g. an enlarged BA pool enriched in MCAs [3, 8]. We hypothesized that high levels of MCAs may be important for the resistance against impaired glucose metabolism and that *Cyp8b1*^*-/-*^ mice therefore should also be resistant to glucose intolerance. Studies were performed on both females and males and the results are based on the combined data, while they persisted after separate analysis. Data on females and males separately is presented in Tables 1 and 2. Of note, some responses to the HFD were even more pronounced in the males, e.g. response to IITT, liver weight and liver triglyceride content.

**Table 1:** Responses to a high fat diet (HFD) in female Cyp8b1-/- mice (ko) and their wildtype (wt) littermates.

FEMALES	wt chow n = 8	ko chow n = 8	wt HFD n = 10	ko HFD n = 9
**AUC Blood glucose during OGTT (mM)**	1399 ±66‡	1329 ±79§	2176±142*	1851 ±63†
**AUC Blood glucose during IITT (mM)**	524 ±31	620 ±96	623 ±58	482 ±42
**Liver weight (g)**	1.1 ±0.1	1.0 ±0.1	1.4 ±0.1§	1.1 ±0.1‡
**Liver cholesterol (mg/liver)**	23 ±2‡	18 ±1	46 ±5*§	22 ±1‡
**Liver triglycerides (mg/liver)**	30 ±2	27 ±2	36 ±3	30 ±2
**VLDL-cholesterol (mM)**	0.03±0.01‡	0.02 ±0.0	0.01 ±0.0*	0.01 ±0.0
**LDL-cholesterol (mM)**	0.08±0.01‡	0.09±0.01§	0.22±0.05*	0.14±0.01†
**HDL-cholesterol (mM)**	0.6 ±0.1	0.5 ±0.0§	0.9 ±0.1	0.9 ±0.1†
**Final body weight (g)**	32±1.5‡	28±1.0§	50±1.3*§	41±3.2†‡
**AUC Body weight (g)**	317 ±15‡	275 ±12§	441 ±12*	372 ±27†
**AUC Body weight gain (g)**	-2.5 ±3.2‡	0.8 ±4.1§	122 ±6*§	83 ±8‡†
**AUC Food efficiency (a.u.)**	-0,0029	0,0009	0,2600	0,1588
**Fecal FFAs (μmol/24h/g BW)**			0,26	0,73
**Fecal cholesterol (μg/24h/g BW)**			7,3	20
**Fecal triglycerides (μg/24h/g BW)**			4,1	2,5
**Serum campesterol/chol (g/mole)**			3.9 ±0.2	n.d.
**Serum GLP-1 (pg/mL)**	58 ±14	69 ±7	39 ±6	44 ±6

Data is shown as mean ±SEM.

* = p<0.05 vs. wt chow

† = p<0.05 vs. ko chow

‡ = p<0.05 vs. wt HFD

§ = p<0.05 vs. ko HFD.

**Table 2:** Responses to a high fat diet (HFD) in male Cyp8b1-/- mice (ko) and their wildtype (wt) littermates.

MALES	wt chow n = 10	ko chow n = 10	wt HFD n = 9	ko HFD n = 9
**AUC Blood glucose during OGTT (mM)**	1353 ±70‡	1202 ±56§	2244 ±220*	1819 ±128†
**AUC Blood glucose during IITT (mM)**	644 ±46‡	566 ±46§	1153 ±80*	919 ±95†
**Liver weight (g)**	1.5 ±0.1‡	1.4 ±0.1	2.4 ±0.3*	1.7 ±0.2
**Liver cholesterol (mg/liver)**	20 ±1‡	21 ±1	51 ±9*	31 ±5
**Liver triglycerides (mg/liver)**	41 ±2‡	37 ±2	61 ±6*	43 ±9
**VLDL-cholesterol (mM)**	0.03 ±0.0‡	0.02 ±0.0§	0.01 ±0.0*	0.01 ±0.0†
**LDL-cholesterol (mM)**	0.07±0.00‡	0.08 ±0.01§	0.27 ±0.04*	0.19 ±0.04†
**HDL-cholesterol (mM)**	0.6 ±0.0‡	0.6 ±0.0§	1.1 ±0.1*	1.1 ±0.1†
**Final body weight (g)**	36±1.5‡	33±1.5§	51±1.8*§	45±3.5†‡
**AUC Body weight (g)**	354 ±13‡	331 ±13	451 ±20*	413 ±35
**AUC Body weight gain (g)**	-8.0 ±3.8‡	-1.3 ±2.9§	92 ±15*	65 ±14†
**AUC Food efficiency (a.u.)**	-0,0077	-0,0013	0,1601	0,1090
**Fecal FFAs (μmol/24h/g BW)**			0,51	0,97
**Fecal cholesterol (μg/24h/g BW)**			16	39
**Fecal triglycerides (μg/24h/g BW)**			1,2	2,6
**Serum campesterol/chol (g/mole)**			3.3 ±0.2	n.d.
**Serum GLP-1 (pg/mL)**	42 ±12	52 ±12	31 ±5	35 ±5

Data is shown as mean ±SEM.

* = p<0.05 vs. wt chow

† = p<0.05 vs. ko chow

‡ = p<0.05 vs. wt HFD

§ = p<0.05 vs. ko HFD.

The present study shows that *Cyp8b1*^*-/-*^ mice have an improved resistance against diet-induced weight gain and to impaired glucose metabolism when challenged with a HFD, as shown from improved blood glucose levels following OGTT and IITT (Fig 1A–1D). However, there were no differences in fasting blood glucose levels between *Cyp8b1*^*+/+*^ and *Cyp8b1*^*-/-*^ mice on chow, although there was a consistent trend for lower blood glucose in *Cyp8b1*^*-/-*^ mice on chow. Further, *Cyp8b1*^*-/-*^ mice on HFD demonstrate a highly improved resistance against liver enlargement, in line with the finding that they did not accumulate cholesterol or TGs in their livers as *Cyp8b1*^*+/+*^ mice on HFD did (Fig 2A–2C). The cause for why liver enlargement did not occur in the *Cyp8b1*^*-/-*^ mice is unclear. It may involve a general reduction in the absorption of dietary fat, but the absence of cholic acid and the high abundancy of FXR antagonistic MCAs [16] may also play a part while these two conditions lower FGF15 expression [17, 18]. A normal FGF15 expression has been reported as crucial for liver regeneration [19].

The HFD also induced a 2-fold increase in serum LDL-cholesterol in *Cyp8b1*^*+/+*^ mice, a response that was dampened by 35% in *Cyp8b1*^*-/-*^ mice, while HDL-cholesterol increased to similar extents in both genotypes. From the initial hypothesis, it was anticipated that a general reduction in the intestinal absorption of fat due to the high abundancy of MCAs, should be important for these responses. In line with this, *Cyp8b1*^*-/-*^ mice on HFD had a doubled fecal excretion of cholesterol and a clear trend for a doubled fecal excretion of FFAs, although the latter did not reach statistical significance. Fecal excretion of TGs was however unaltered. Thus, these data together with observations by other research groups [6, 20] strongly suggest that lipid absorption is impaired in *Cyp8b1*^*-/-*^ mice. Therefore, a reduction in lipid absorption is likely to in part explain why *Cyp8b1*^*-/-*^ mice are more resistant to HFD-induced weight gain, steatosis, and to glucose intolerance. Since the BA level and composition is strongly altered in *Cyp8b1*^*-/-*^ mice there is also reason to expect that there may be changes in the basal metabolic rate while BAs have been shown to influence that [21]. Further studies thus need to thoroughly monitor oxygen consumption, carbon dioxide production, body temperature, and physical activity as well as intestinal transit in *Cyp8b1*^*-/-*^ mice. As mentioned, the phenotype of *Cyp8b1*^*-/-*^ mice has similarities with that of germ-free mice [12] e.g. similar BA composition [3, 6, 16] and reduced absorption of cholesterol [12]. As for the latter, this could be due to an impaired ability of MCAs to form micelles as discussed by Wang et al. [2].

The mechanism for why germ-free mice are resistant to develop glucose intolerance has been suggested to be linked to the absence of microbiota. The current results showing that also *Cyp8b1*^*-/-*^ mice are resistant to glucose intolerance, suggest that the very absence of a microbiota may not be key, lending support for the possibility that altered BA level and composition could be important since BAs regulate the microbiota [22]. Assessments of the microbial composition in *Cyp8b1*^*-/-*^ mice are thus another aspect to include in future studies.

Interestingly, Kaur et al. found that GLP-1 levels are increased in chow-fed *Cyp8b1*^*-/-*^ mice, which was suggested to explain why these animals have lower blood glucose levels [20]. In the present study, *Cyp8b1*^*-/-*^ mice on chow showed a strong trend for reduced blood glucose, although this did reach statistical significance. This discrepancy may be due to that the mice in their study were younger than in the present study (i.e. 3–6 vs 10–11 months). In the current study, GLP-1 was measured in 4h-fasting serum samples from the time of sacrifice. This was a suboptimal time point to evaluate the postprandial hormone GLP-1. Nevertheless, in line with Kaur et al., GLP-1 levels tended to be higher in *Cyp8b1*^*-/-*^ mice than in *Cyp8b1*^*+/+*^ mice on chow. Exposure to HFD reduced GLP-1 levels in both genotypes but to a lesser extent in *Cyp8b1*^*-/-*^ mice. This may in part explain why HFD induces glucose intolerance and insulin resistance in mice as well as why *Cyp8b1*^*-/-*^ mice are better protected. Further, it has been reported that in mice where the energy supply is limited, increased basal circulating GLP-1 from colon reduces intestinal transit thereby promoting absorption from the intestinal lumen to compensate for the limited energy supply [23]. It remains to be evaluated whether this explains why circulating GLP-1 levels are increased in *Cyp8b1*^*-/-*^ mice. One may speculate if the increased circulating GLP-1 levels indicate that the energy supply in colon is limited in *Cyp8b1*^*-/-*^ mice. Clearly, tuning of intestinal absorption is complex and certainly warrants further investigation in *Cyp8b1*^*-/-*^ mice. Such studies are now under way to better understand the phenotype of *Cyp8b1*^*-/-*^ mice.

In summary, these findings reveal that *Cyp8b1*^*-/-*^ mice with a BA pool abundant in MCAs are protected against the development of several stigmata of the metabolic syndrome such as body and liver weight gain, steatosis, hypercholesterolemia and insulin resistance. The results indicate that MCAs may be of therapeutic potential for treatment of metabolic syndrome.
